# Enhancing Cardiovascular Health and Functional Recovery in Stroke Survivors: A Randomized Controlled Trial of Stroke-Specific and Cardiac Rehabilitation Protocols for Optimized Rehabilitation

**DOI:** 10.3390/jcm12206589

**Published:** 2023-10-18

**Authors:** Moattar Raza Rizvi, Ankita Sharma, Ahmed Malki, Waqas Sami

**Affiliations:** 1 Department of Physiotherapy, School of Allied Health Sciences, Manav Rachna International Institute of Research and Studies (MRIIRS), Faridabad 121001, India; mrrizvi.fas@mriu.edu.in (M.R.R.); ankitasharma.fas@mriu.edu.in (A.S.); 2 Department of Biomedical Sciences, College of Health Sciences, QU-Health, Qatar University, Doha P.O. Box 2713, Qatar; ahmed.malki@qu.edu.qa; 3 Department of Pre-Clinical Affairs, College of Nursing, QU Health, Qatar University, Doha P.O. Box 2713, Qatar

**Keywords:** stroke, cardiac rehabilitation, exercise, balance, mobility, cardiovascular fitness, respiratory performance, autonomic function

## Abstract

Background: Stroke is a major contributor to disability and mortality globally. It leads to physical impairments, including weakness and cardiovascular deconditioning, posing significant challenges to stroke survivors’ quality of life. Exercise-based cardiac rehabilitation has shown promise as a rehabilitation strategy. This study aims to assess and compare the impacts of stroke-specific rehabilitation and individualized cardiac rehabilitation exercises on various health parameters in stroke patients. Methods: A randomized controlled trial was conducted, involving 38 stroke patients aged 40–75 years. Group A received stroke-specific rehabilitation, which consisted of a combination of range of motion exercises, strength training for the paralyzed side, balance and coordination training, gait training, functional mobility exercises, neuromuscular reeducation, and breathing exercises. This program was conducted five days per week for 12 weeks. Group B received individually designed cardiac rehabilitation exercises, in addition to stroke-specific rehabilitation. They engaged in this exercise for at least 30–45 min per day, four days per week, and incorporated two days of resistive training over a 12-week period. Baseline and post-intervention assessments included measures of cardiac autonomic function, balance (Berg Balance Scale), mobility (Timed Up and Go Test), cardiovascular fitness indicators, respiratory parameters, exercise efficiency, and perceived exertion. Results: Group B receiving individualized cardio rehab showed significant improvements in balance and mobility compared to Group A receiving conventional stroke-specific rehab. Moreover, Group B exhibited enhanced cardiovascular fitness, respiratory performance, exercise efficiency, and autonomic function post-intervention. Notably, Group A displayed no significant improvements in these parameters. Conclusions: Individualized cardiac rehabilitation exercises demonstrated favorable outcomes in improving certain health parameters, highlighting the potential benefits of individualized rehabilitation strategies for stroke patients.

## 1. Introduction:

Stroke is a leading cause of long-term disability and mortality worldwide, posing a significant burden on individuals and healthcare systems [1,2]. It occurs when the blood supply to the brain is disrupted, either by a blockage (ischemic stroke) or bleeding (hemorrhagic stroke), leading to the death of brain cells [3]. The resulting impairments can have a profound impact on physical function and overall quality of life for stroke survivors. Physical function encompasses various domains, including endurance, strength, balance, coordination, and motor control. These impairments and disabilities increased demand for tailored rehabilitation programs to improve recovery and quality of life.

Cardiorespiratory endurance, an important component of physical fitness, is often compromised due to reduced physical activity levels and impaired cardiovascular function after stroke [4]. Autonomic dysfunction, characterized by disrupted regulation of the autonomic nervous system, can manifest as abnormal heart rate variability, altered blood pressure control, and increased sympathetic activity. These dysfunctions contribute to a higher risk of secondary cardiovascular events and further compromise functional capacity and quality of life in stroke survivors [5].

Exercise-based cardiac rehabilitation, which has traditionally been employed in individuals with cardiovascular diseases, has emerged as a promising approach for stroke rehabilitation and stroke prevention. This rehabilitation modality typically combines aerobic exercise, resistance training, and education on risk factor management [6,7]. Nevertheless, the effects of cardiovascular training in the early stages of stroke are not fully investigated.

The rationale for evaluating exercise-based cardiac rehabilitation in stroke patients is multifaceted. Firstly, exercise interventions have been shown to improve cardiovascular fitness, muscle strength, and functional capacity in various populations, including individuals with cardiovascular diseases [8]. Secondly, regular physical activity can stimulate the reorganization of neural networks and promote the formation of new connections, potentially enhancing motor function [9]. Thirdly, exercise-based cardiac rehabilitation programs typically incorporate comprehensive assessments of endurance, functional strength, and autonomic function outcomes [10]. 

There are different types of exercise programs that stroke patients can participate in, including conventional exercise and cardio rehab [11]. Stroke-specific rehab typically involves range of motion exercises, strength training for paralyzed side, balance and coordination training, gait training, functional mobility exercises, neuromuscular reeducation, and breathing exercises [12]. These exercises are designed to improve muscle strength, muscle tone, balance, and coordination. They are usually performed under the supervision of a physical therapist and are generally safe and effective for stroke patients, but they may not be suitable for patients with severe disabilities [13,14]. Cardio rehab programs are usually more intensive than stroke-specific rehab. However, they may be more effective for stroke patients who have severe disabilities or who need intensive rehabilitation [15].

Physical activity and exercise recommendations for stroke survivors have been extensively studied [16]. The American Heart Association recommends that stroke survivors engage in moderate-intensity aerobic exercise for at least 20–30 min per day, five days per week, or vigorous-intensity aerobic exercise for at least 25 min per day, three days per week. Strength training exercises should also be performed two to three times per week, and flexibility exercises should be completed daily. These recommendations are based on the evidence that physical activity and exercise can improve cardiovascular health, reduce the risk of future strokes, and improve physical function in stroke survivors [9].

Analyzing heart rate variability (HRV) in the chronic phase of stroke offers valuable insights into predicting stroke recurrence, mortality, and potential underlying mechanisms [17]. Utilizing HRV parameters as biomarkers for stroke necessitates a comprehensive approach that combines both linear and nonlinear methods [18]. This study aimed to investigate and compare the effectiveness of two distinct rehabilitation protocols: stroke-specific rehabilitation and individualized cardiac rehabilitation exercises. The study evaluated a range of outcome measures, including cardiac autonomic function, balance, mobility, cardiovascular fitness, respiratory parameters, and exercise efficiency. The primary objective of this study is to assess the impact of these interventions on enhancing cardiac recovery in individuals undergoing stroke rehabilitation.

## 2. Methodology

### 2.1. Study Design

A randomized controlled trial was conducted to assess the impact of two distinct rehabilitation approaches among stroke patients. The study consisted of two groups: Group A received stroke-specific rehabilitation protocol, and Group B received a combined intervention of individualized cardiac rehabilitation in addition to stroke rehabilitation. Single blinding was implemented to the extent possible given the nature of the interventions. While complete blinding of participants, therapists, and assessors was not feasible due to the distinct rehabilitation approaches involved, efforts were made to minimize potential biases. Group assignment was concealed until after baseline assessments were completed to reduce selection bias. Assessors responsible for collecting outcome data were blinded to group allocation to minimize measurement bias.

### 2.2. Sample Size Calculation

G*Power (version 3.1.9.4) was utilized to calculate the sample size for this study. An a priori power analysis was conducted for a repeated-measures analysis of variance with 80% power (α = 0.05) to detect a medium effect (η^2^p = 0.05), focusing on examining main effects and interactions between two groups with two repeated measures in our dependent measures of interest. Based on the power analysis, it was determined that a total of 34 participants would be needed to achieve the desired statistical power. However, taking into account an estimated dropout rate of 10%, the study was initiated with a sample size of 38 participants. By considering the potential dropout rate and starting with a slightly larger sample size, the study aims to ensure that enough data were available for analysis, even if some participants withdraw from the study during its course.

### 2.3. Sampling Technique

In this study, we utilized computerized random sampling to select participants. This method involved employing computer software to generate random numbers or sequences, which were then used to determine the assignment of participants to either Group A or Group B. Computerized random sampling offers a highly systematic and unbiased approach to participant selection, further enhancing the study’s methodological rigor and minimizing potential biases in group assignment.

### 2.4. Participants

**Figure 1:**
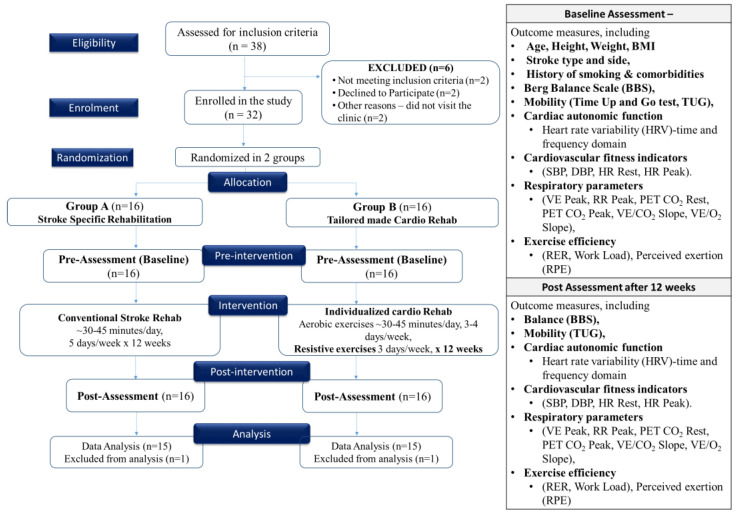
Consort diagram and study flowchart.

Stroke patients aged 40–75 years were recruited from rehabilitation centers or hospitals. They were randomly assigned to either Group A or Group B. Group A participated in a structured stroke rehabilitation protocol, which involved daily sessions conducted five days a week over a period of 12 weeks. In contrast, Group B received a combined intervention, which consisted of individualized cardiac rehabilitation in addition to stroke rehabilitation. This intervention included approximately 30–45 min of moderate-intensity aerobic exercise per day, spanning four days a week, along with two days of whole-body resistance-training exercises each week, all administered over a duration of 12 weeks. Baseline assessments at 0 day and post-intervention assessment after 12 weeks were conducted for all participants to measure cardiac autonomic function, balance, mobility, cardiovascular fitness, respiratory parameters, and exercise efficiency. The program was aimed to improve cardiovascular fitness, respiratory parameters, endurance, and autonomic function (Figure 1).

### 2.5. Inclusion Criteria

Inclusion criteria included patients with confirmed medical diagnosis of ischemic stroke, age between 40–75 years, in the subacute stage of ischemic stroke according to the Burnstrom classification (1 to 3 months post-stroke), medically stable and cleared for exercise by a healthcare professional [19]. The study primarily concentrated on male participants to improve its generalizability given the higher incidence of ischemic stroke in males [20]. To be considered for the program, participants must demonstrate the ability to walk independently for a distance exceeding 10 m, with or without the use of mobility aids. Additionally, they must have a Chedoke–McMaster Stroke Assessment leg impairment score of 3 or higher, indicating the presence of marked spasticity and weakness, or a lower degree of impairment [21].

### 2.6. Exclusion Criteria

Patients with severe cognitive impairments, uncontrolled medical conditions (such as cardiac or respiratory diseases, hypertension, or active infections), and contraindicated for maximal exercise testing (including recent myocardial infarction, unstable angina, severe arrhythmias, uncontrolled heart failure, or severe orthopedic conditions), significant neurological impairments, severe comorbidities, conditions limiting exercise safely, inability to provide informed consent, or non-compliance with study procedures were excluded. 

### 2.7. Outcome Measures Evaluation

The methodology employed in this study encompassed a comprehensive assessment of various outcome measures, including assessment of balance using the Berg Balance Scale (BBS), mobility using the Timed Up and Go Test (TUG), and cardiac autonomic function using heart rate variability (HRV). Furthermore, cardiovascular fitness indicators such as peak oxygen consumption (VO_2_ Peak), peak oxygen pulse (O_2_ Pulse Peak), resting heart rate (HR Rest), and peak heart rate (HR Peak) and respiratory parameters such as peak minute ventilation (VE Peak), peak respiratory rate (RR Peak), resting end-tidal carbon dioxide (PET CO_2_ Rest), peak end-tidal carbon dioxide (PET CO_2_ Peak), ventilatory equivalent for carbon dioxide (VE/CO_2_ Slope), ventilatory equivalent for oxygen (VE/O_2_ Slope), exercise efficiency (respiratory exchange ratio, RER and workload), and perceived exertion (RPE) were also examined. 

For evaluating balance, the Berg Balance Scale (BBS) was utilized, consisting of tasks that participants performed to assess their balance and stability [22]. The scale provides scores ranging from 0 to 56, with higher scores denoting better balance. Mobility was assessed through the Timed Up and Go Test (TUG), where participants were timed as they stood up, walked a distance of three meters, turned around, returned, and sat down. The time taken to complete this task was recorded as a measure of mobility [23].

Cardiac autonomic function was evaluated by analyzing heart rate variability (HRV) from electrocardiogram (ECG) data. Time domain measures such as SDNN and RMSSD, as well as frequency domain components like LF (Low Frequency) and HF (High Frequency), were examined as previously described [24]. Furthermore, cardiovascular fitness indicators, including systolic blood pressure (SBP), diastolic blood pressure (DBP), resting heart rate (HR Rest), and peak heart rate (HR Peak), were measured. SBP and DBP represented arterial blood pressure during rest, while HR Rest and HR Peak were assessed at rest and after maximal exercise, respectively [25]. Respiratory parameters, such as peak minute ventilation (VE Peak), peak respiratory rate (RR Peak), resting end-tidal CO_2_ pressure (PET CO_2_ Rest), and peak end-tidal CO_2_ pressure (PET CO_2_ Peak), were measured with the use of spirometer. These parameters provided insights into respiratory responses during both resting and maximal exercise conditions. Additionally, the VE/CO_2_ Slope and VE/O_2_ Slope were calculated to assess gas exchange efficiency during exercise [26].

Exercise efficiency was evaluated through the respiratory exchange ratio (RER), indicating the type of fuel source (carbohydrates vs. fats) used during exercise, and the measurement of workload, typically quantified in watts or other units, to reflect the workload achieved during exercise testing. Participants also reported their perceived exertion levels using the Rate of Perceived Exertion (RPE) scale, specifically the Borg Rating of Perceived Exertion [27]. 

### 2.8. Stroke-Specific Rehabilitation Protocol 

A combination of range of motion exercises, strength training, balance and coordination training, gait training, functional mobility exercises, neuromuscular reeducation, and breathing exercises all were given to both the groups to improve functional abilities after stroke [28]. The patients were monitored completely throughout the exercises and the protocol was changed depending upon the individual’s response and ability to complete exercises. The supervised exercise program was administered for 5 days a week for 12 weeks. Additionally, the patients were counselled to remain active and to include 30–40 min of any aerobic exercise of their choice to be completed at home and to record the same in their exercise dairy.

### 2.9. Individualized Cardiac Rehabilitation Exercise Protocol

Along with the stroke-specific rehab and keeping in mind the standard cardiac rehabilitation exercise protocol, each patient’s training protocol was individualized with progressions. The exercises included resistance training and resistive exercises. The aerobic training was administered for 3–4 days in a week and resistance training for whole body was administered 2 times in a week. All the exercises were completed in the active supervision of the therapist, and they were stopped any time the patient felt uncomfortable. The following guidelines elucidate the exercise administration process.

Aerobic training was administered five days a week, with exercise intensity set at 60–80% of heart rate reserve (HRR) [29]. Furthermore, exercise intensity was consistently kept below 12 to 14 on the rating of perceived exertion (RPE) scale, as pre-calculated. Throughout the program, heart rate was closely monitored, and any aerobic activity was promptly ceased if heart rate exceeded 60–65% of maximum heart rate (HR max).

Aerobic exercise sessions were extended to last between 30 and 45 min. Exercise intensity was monitored using the final HR and RPE taken at the conclusion of the training session. The weekly average training HR was calculated based on the final HR recordings across the entire session. The exercise modality was personalized for each individual. Patients capable of walking at sufficient speed and duration to achieve aerobic benefits were prescribed treadmill walking. Upright and semi-recumbent cycle ergometers were also incorporated into the program. Patients unable to sustain a faster walking or cycling pace for extended durations were also offered interval training. This consisted of short bouts of higher-intensity exercise followed by longer bouts of lower-intensity exercise. Priority was placed on the total duration of exercises rather than the type of exercise performed.

A resistance-training program with an initial weight load of 60% of the 1-repetition maximum for non-paretic limbs and a rating of 11–14% for paretic limbs was administered. This program included 7–10 upper and lower body exercises. Participants began with 10 repetitions and gradually progressed to 15 repetitions before increasing their weights. Various resistance tools such as dumbbells, resistance bands, body weight, and weight machines were utilized. The training regimen was based on individual assessments of functional abilities, hypertonicity, range of motion, and balance impairment. The total duration of exercise was kept between 60 and 75 min per day with a gap of 20 min between the stroke-specific exercises and exercises focusing on cardiac rehabilitation.

### 2.10. Data Collection and Analysis

Statistical analysis was conducted using SPSS Version 22. Prior to performing parametric tests, we assessed the normality assumption using the Shapiro–Wilk test. Follow-up assessments were performed after the completion of the exercise-based cardiac rehabilitation program or an equivalent duration for the control group. To analyze the data between two groups, an independent *t*-test was employed, followed by a paired *t*-test for within-group analysis. A 95% confidence interval and a significance level of *p* < 0.05 were used. Descriptive statistics were utilized to calculate and report the mean and standard deviation of participants in both Group A and Group B.

## 3. Results

In this study, we conducted a comprehensive assessment of demographic and medical factors in stroke patients categorized in two distinct groups, Group A [n = 15; male = 12 (80%) and female = 3 (20%)] and Group B [n = 15; male = 13 (86.7%) and female = 2 (13.3%)]. Both groups exhibited similar mean ages (Group A: 59.60 ± 2.72; Group B: 60.40 ± 3.20 years; t = −0.74, *p* = 0.47). Comparable measurements were observed for height (Group A: 1.47 ± 0.44; Group B: 1.45 ± 0.43 m, t = 1.22, *p* = 0.23), weight (Group A: 65.20 ± 9.72; Group B: 64.80 ± 10.21 kg, t = 0.11, *p* = 0.91), and BMI (Group A: 30.84 ± 1.79; Group B: 30.62 ± 4.12, t = 0.19, *p* = 0.85). Investigation into stroke type distribution demonstrated that 86.7% of Group A and 73.3% of Group B had ischemic strokes (χ^2^ = 0.83, *p* = 0.36), without significant differences. Similarly, no significant distinctions were noted in terms of stroke side, with 73.3% left-sided strokes in Group A and 80.0% in Group B (χ^2^ = 0.19, *p* = 0.67). Notably, there was a notable divergence in smoking habits, with 66.7% smokers in Group A and 93.3% in Group B (χ^2^ = 3.33, *p* = 0.17), but without any significance. Rates of hypertension were 66.7% in Group A and 73.3% in Group B (χ^2^ = 0.16, *p* = 0.69), showing no significant difference. Additionally, diabetes exhibited a prevalence of 93.3% in Group A and 80.0% in Group B (χ^2^ = 1.15, *p* = 0.28), without statistical significance. Likewise, heart failure was present in 80.0% of Group A and 73.3% of Group B (χ^2^ = 0.19, *p* = 0.67), demonstrating no significant variation between the two groups. These findings provide valuable insights into the baseline characteristics of our study population (Table 1).

Further, independent *t*-test was used to compare the outcomes between Group A and Group B for the Berg Balance Scale (BBS) and Timed Up and Go (TUG) tests. In the independent *t*-test analysis comparing Group A and Group B, we assessed various measures related to balance and mobility. In terms of the BBS, there was no significant difference in pre-intervention scores (Group A: 39.67 ± 4.79, Group B: 38.13 ± 2.45; t = 1.10, *p* = 0.28) or post-intervention scores (Group A: 41.73 ± 3.56, Group B: 42.73 ± 3.69; t = −0.76, *p* = 0.46) between the two groups. Similarly, for the TUG, there were no significant differences in pre-intervention scores (Group A: 26.93 ± 3.06, Group B: 25.93 ± 2.05; t = 1.05, *p* = 0.30). However, post-intervention TUG scores showed a significant improvement between Group A (23.60 ± 3.36) and Group B (20.53 ± 3.04; t = 2.62, *p* = 0.01), with a mean difference of 2.69 s (95% CI: 0.67–5.46).

Paired *t*-tests were used to examine specific variables within each group before and after the intervention. Group A demonstrated a minimal change in BBS scores from pre- to post-intervention, with scores of 39.67 ± 4.79 and 41.73 ± 3.56, respectively (t = −1.20, *p* = 0.25), indicating only a slight increase in scores by 2.07. In contrast, Group B exhibited a notable improvement in BBS scores following the intervention, with scores changing from 38.13 ± 2.45 to 42.73 ± 3.69 (t = −3.83, *p* = 0.002). This indicates a substantial increase in scores by 4.60. Group A displayed no improvement in TUG scores post-intervention, with times decreasing from 26.93 ± 3.06 to 23.60 ± 3.36 (t = 26.46, *p* = 0.67). This signifies a substantial reduction in time by 3.33. Similarly, Group B exhibited a significant enhancement in TUG scores, with times decreasing from 25.93 ± 2.05 to 20.53 ± 3.04 (t = 7.28, *p* < 0.001), representing a considerable reduction in time by 5.40.

Table 2 presents the results of an independent *t*-test comparing the effects of stroke-specific rehab in Group A and individualized cardio rehab in Group B. Group B exhibited significantly improved Post-Mean NN (*p* = 0.01) and Post RMSSD (*p* = 0.01) compared to Group A, indicating enhanced heart rate variability and parasympathetic nervous system activity following the intervention. However, there were no significant differences between the groups in Pre Mean NN, Resting Pre HR, Resting Post HR, Pre SDNN, Post SDNN, Pre NN50, Pre LF, Post LF, Pre HF, Pre VLF, Post VLF, Pre TP, Post TP, Pre LF/HF, Pre nLF, and Post nLF (all *p* > 0.05).

The results for cardiovascular fitness indicators (SBP, DBP, HR Rest, HR Peak) showed no significant differences between Group A and Group B in both Pre and Post measurements (*p* > 0.05), indicating similar baseline characteristics and responses to the interventions (Table 2). For respiratory parameters, there were significant differences in VE Peak, RR Peak, PET CO_2_ Peak, VE/CO_2_ Slope, and VE/O_2_ Slope between the groups post-intervention (*p* < 0.05), suggesting that Group B had improved respiratory performance compared to Group A. Additionally, Group B exhibited a significant decrease in RER and a significant increase in exercise duration compared to Group A post-intervention (*p* < 0.05), indicating enhanced exercise efficiency and endurance in Group B (Table 3).

The paired *t*-test results reveal noteworthy differences between Group A and Group B in various heart rate variability (HRV) and spectral domain parameters following the intervention (Table 4). Group B exhibited significant improvements (*p* < 0.05) in HR Rest, SDNN, RMSSD, NN50, VLF, and LF/HF compared to Group A. These improvements suggest that the intervention received by Group B had a positive effect on their cardiac autonomic function and vagal tone, likely contributing to better overall cardiovascular health.

**Figure 2:**
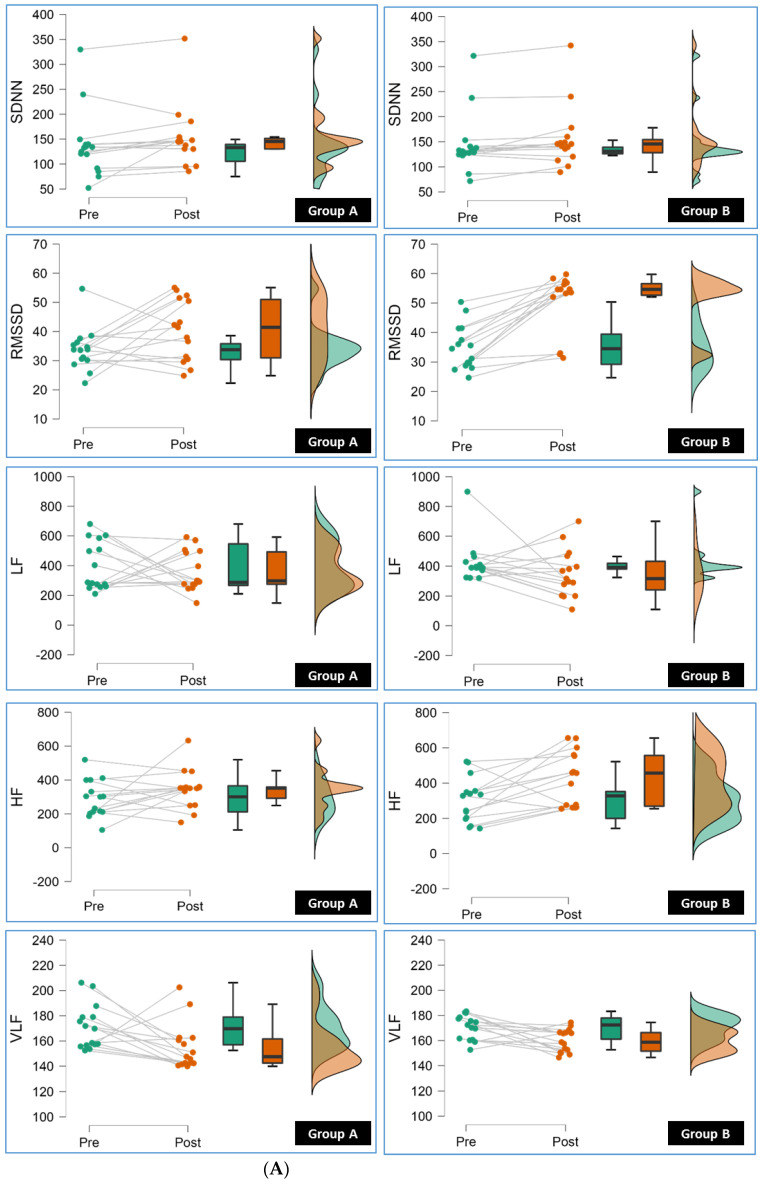

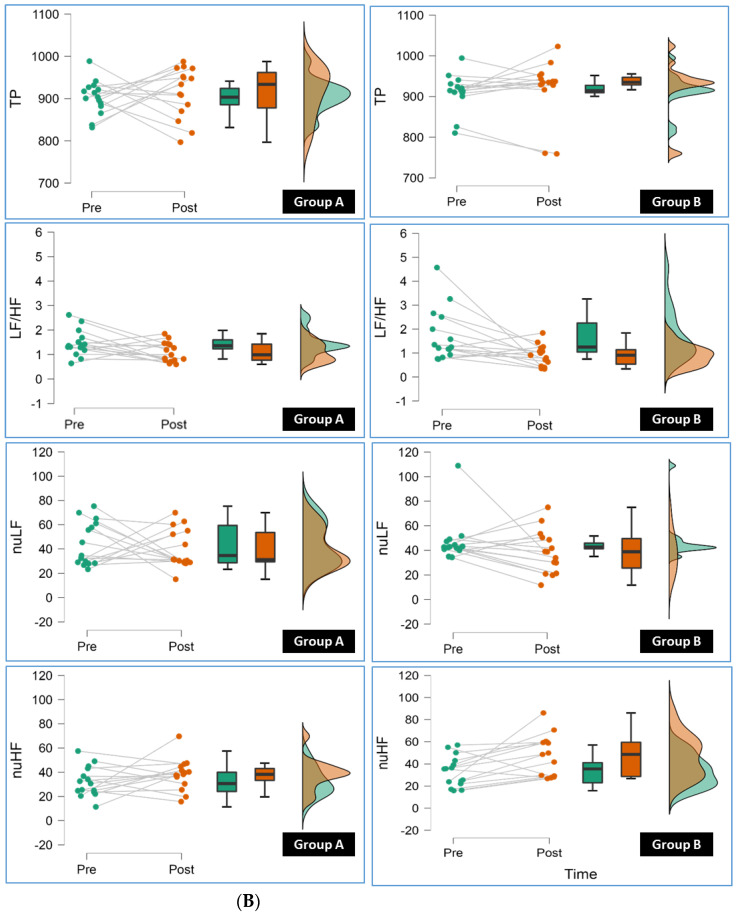
(**A**): The line, box, and density plots from repeated-measure ANOVA in this raincloud display the pre to post measurement of SDNN (Standard Deviation of NN intervals), RMSSD (Root Mean Square of Successive Differences), LF (Low Frequency), HF (High Frequency), VLF (Very Low Frequency) for individual participants in Group A and Group B (pre and post). (**B**): The line, box, and density plots from repeated-measure ANOVA in this raincloud display the pre to post measurement of TP (Total Power), LF/HF (Low Frequency to High Frequency Ratio), nLF (Normalized Low Frequency), nHF (Normalized High Frequency) for individual participants in Group A and Group B.

Conversely, Group B also showed significant reductions (*p* < 0.05) in HR Peak, LF, and nHF compared to Group A. These reductions imply favorable changes in HRV parameters associated with reduced sympathetic activity, which is generally considered beneficial for cardiovascular health. Figure 2A,B show the results of repeated-measure ANOVA conducted for Group A and Group B.

In analyzing cardiovascular fitness indicators, it is noteworthy that Group A exhibited a non-significant change in systolic blood pressure (SBP) but demonstrated a significant reduction in heart rate at peak exertion (HR Peak; Table 5). Conversely, Group B displayed a significant decrease in SBP and resting heart rate (HR Rest), as well as a significant reduction in HR Peak. Both groups had non-significant changes in diastolic blood pressure (DBP).

Shifting the focus to respiratory parameters, both Group A and Group B showed non-significant changes in peak minute ventilation (VE Peak), peak respiratory rate (RR Peak), and resting and peak end-tidal carbon dioxide levels (PET CO_2_ Rest and PET CO_2_ Peak). However, Group B demonstrated significant improvements in ventilatory efficiency, as evidenced by a significant reduction in the ventilatory equivalent for carbon dioxide (VE/CO_2_ Slope) and improved oxygen consumption efficiency, reflected in the significant decrease in the ventilatory equivalent for oxygen (VE/O_2_ Slope). In contrast, Group A had non-significant changes in these parameters.

Considering exercise efficiency, both groups displayed significant decreases in the respiratory exchange ratio (RER), suggesting improved substrate utilization during exercise. However, there were no significant changes in workload for either group. When evaluating perceived exertion (RPE) and exercise duration, both Group A and Group B demonstrated non-significant changes.

## 4. Discussion

Stroke patients often experience a myriad of physical and physiological challenges, requiring comprehensive rehabilitation strategies to restore their overall health and quality of life [30]. Aerobic exercise options for post-stroke rehabilitation are diversifying, including overground walking, treadmill training, sports, and technology-based solutions [29]. Stroke patients unable to walk independently may benefit from electromechanical-assisted gait training, possibly involving body weight support [31]. The effectiveness of mental practice for enhancing stroke patient recovery varies, with mixed findings in different studies [32]. This study seeks to address the critical question of whether individually designed cardiac rehabilitation programs specific to the needs of stroke patients offer superior outcomes compared to stroke-specific rehab, which targets the major functional limitations a patient has following a stroke.

In this study, the stroke patients who underwent individualized cardio rehab experienced notable enhancements in balance and mobility, two critical components of post-stroke rehabilitation and overall well-being. These exercises specifically address muscle strength, proprioception, spatial awareness, and cardiovascular health—key factors in the recovery process for stroke survivors. The Berg Balance Scale (BBS) and Short Physical Performance Battery (SPPB) emerged as effective assessment tools for balance and mobility in stroke patients [33]. A systematic review confirmed the positive effects of exercise programs on physical function, strength, and daily activities in stroke patients, emphasizing the importance of tailoring exercise regimens based on the stage of stroke [34]. The American Heart Association also recommends regular physical activity and exercise for stroke survivors, encompassing aerobic exercise, strength training, and balance training to enhance physical function and overall health [9].

Autonomic disturbances following a stroke are a common occurrence but have often been inadequately understood and researched [35]. One contributing factor to this lack of comprehensive study is the intricate nature of the autonomic nervous system (ANS). The ANS operates with a complex hierarchy, intricate connections, and multiple control points, making it challenging to isolate the specific effects of individual pathways [36]. Moreover, evaluating the ANS in a clinical context is constrained as existing tests are primarily designed for research, rendering them costly and less accessible. Various confounding factors, including underlying medical conditions, medication usage, hydration status, and extended severe disability, can also influence test outcomes. Following individualized cardio rehab exercises in the present study, stroke patients exhibited significant improvements in several key heart rate variability (HRV) and spectral domain parameters, shedding light on the underlying mechanisms of these changes.

The previous study found that elevated resting heart rate (RHR) was not significantly associated with the overall risk of stroke. However, this association appeared to be significant among men [37]. In another prospective study involving a large Chinese adult population aged 40 and older, it was observed that a higher RHR increased the risk of total and hemorrhagic strokes but not ischemic stroke [38]. This comprehensive meta-analysis revealed that, for every 10 beats per minute increase in RHR, there was a 6% higher risk of stroke. Furthermore, individuals with an RHR exceeding 80 bpm had a 47% greater risk of hemorrhagic stroke, a 38% higher risk of ischemic stroke, and a 68% elevated risk of unclassified stroke compared to those with an RHR below 65 bpm. In the present study, the reduction in resting heart rate (HR Rest) indicates enhanced heart efficiency, which is often associated with improved cardiovascular fitness [38].

The increased NN intervals (SDNN) and root mean square of successive differences (RMSSD) in stroke patients receiving individualized cardio rehab suggest better autonomic regulation and improved cardiac adaptability to stress. Heart rate recovery, or how quickly the heart rate returns to normal after exercise, is a crucial sign of cardiac adaptability. Regular exercise can help with heart rate recovery as part of cardiac rehabilitation, which may be shown in higher SDNN and RMSSD [39]. An individualized rehabilitation program featuring a gradual progression of exercises enables patients to acclimate to rising levels of physical activity over time. This controlled approach can be the reason for the adaptation of the cardiovascular system and enhancement of HRV parameters [40]. Exercise training can positively influence autonomic function and contribute to quicker HRR recovery after exertion [41]. It is important to note that, while increased SDNN and RMSSD are generally positive indicators of improved autonomic regulation and cardiac adaptability, individual responses to cardiac rehabilitation can vary [42]. Therefore, the specific reasons for these improvements may vary from patient to patient [43].

Elevated very low-frequency power (VLF) suggests enhanced thermoregulation and potentially reduced renin–angiotensin–aldosterone system activity, which can positively influence blood pressure regulation and overall cardiovascular function. Furthermore, the VLF band has a stronger association with cardiovascular disease prognosis, metabolic syndromes, and all-cause mortality after traumatic brain injury than with the other HRV components [44]. Furthermore, the favorable shift in the low-frequency to high-frequency ratio (LF/HF ratio) indicates improved autonomic balance and stress response [45]. These findings collectively suggest that individualized cardio rehab exercises have a profound positive impact on cardiac autonomic function, sympathetic–parasympathetic balance, thermoregulation, and renin–angiotensin–aldosterone system activity in stroke patients, contributing to their enhanced cardiovascular health [46].

The individualized cardiac rehab program has demonstrated significant improvements in ventilatory efficiency, reflected by the significant decrease in the ventilatory equivalent for oxygen (VE/O_2_ Slope) as compared to group A. VE/O_2_ can be used to monitor changes in exercise tolerance and efficiency over time [47]. Patients with a stroke may experience respiratory muscle paralysis or dysfunction, which can hinder their capacity to breathe effectively during physical activity. A high VE/O_2_ ratio may indicate respiratory limitations [48]. This can serve as a guide for interventions, such as respiratory muscle training, to enhance ventilation efficacy. Cardiac rehabilitation can enhance cardiac function, including increased stroke volume and cardiac output when a patient exercises at moderate intensity. As the heart pumps more efficiently, the body receives an adequate oxygen supply with fewer ventilatory efforts, reducing the VE/O_2_ Slope [49]. Aerobic exercise programs often target respiratory muscles as they require an increased respiratory rate during exercise. Improved strength and endurance of these muscles can contribute to more efficient ventilation and oxygen exchange [6].

Our study, which centered on individualized cardio rehab for stroke patients, revealed non-significant reductions in the respiratory exchange ratio (RER), pointing to improved exercise efficiency with enhanced substrate utilization. Moreover, we observed a decrease in the rating of perceived exertion (RPE), highlighting that participants found the exercise less strenuous. These combined outcomes suggest that personalized rehabilitation programs can effectively enhance both physiological and subjective aspects of exercise tolerance in stroke patients, offering a comprehensive approach to their recovery. RPE measurement under sub-maximum exercise conditions was well correlated with other established physical fitness indicators in both trained and untrained men, suggesting that RER can be used as a measure of exercise efficiency in people with reduced exercise tolerance [27]. In longitudinal studies, a decrease in RER has been observed after training at the same absolute workload but not at the same relative intensity, indicating improved substrate utilization during exercise [50]. A study on cardiac rehabilitation in patients with coronary artery disease found that a lower RER peak might not reach the peak exercise tolerance value, and few research studies have investigated the influence of low peak RER on cardiac rehabilitation [51]. Another study found that the oxygen consumption and cardiorespiratory load during robot-assisted gait after stroke is low, indicating a decrease in RPE [52].

This study has several potential limitations. Firstly, the sample size, although accommodating an estimated dropout rate, remains relatively small, potentially limiting statistical power and generalizability. Additionally, the use of convenience sampling introduces selection bias, and the primary focus on male participants may restrict the applicability of the findings to a broader stroke patient population. Furthermore, the study’s specific focus on ischemic stroke patients in the subacute stage raises questions about generalizability to other stroke types. The lack of control for participants’ medication usage and underlying medical conditions, 12-week follow-up period, and the single-center study design further constrain the study’s scope. Self-reported measures and differences in exercise intensity between the two groups add to the complexity of interpreting the results. Finally, uncontrolled external factors, such as adherence to exercise programs and lifestyle changes outside the study, could introduce confounding variables. These limitations should be considered when interpreting the study’s findings, highlighting the need for further research with larger, more diverse samples and longer-term follow-up.

In the future, several research directions in stroke rehabilitation and tailored exercise interventions merit exploration. Expanding randomized controlled trials to include a more diverse pool of participants, spanning both ischemic and hemorrhagic stroke patients, can provide a broader perspective on the impact of personalized cardiac rehabilitation. Investigating the potential of technology-assisted rehabilitation, such as virtual reality training and electromechanical-assisted gait training, is promising, particularly for those with severe mobility limitations. Further studies on the role of biomarkers as indicators of stroke recovery and treatment response can deepen our understanding of individualized cardio rehab’s underlying mechanisms. Exploring how specific medications and underlying medical conditions interact with exercise interventions may offer valuable insights into tailoring rehabilitation programs to various patient profiles. Lastly, research on the relationship between exercise efficiency, substrate utilization, and perceived exertion in response to different exercise parameters can refine customized exercise regimens to cater to individual stroke patients.

## 5. Conclusions

In conclusion, this study underscores the potential benefits of individualized cardiac rehabilitation programs for stroke patients. By addressing the unique needs of individuals recovering from stroke, these personalized programs have shown significant improvements in balance, mobility, exercise efficiency, and autonomic regulation, as indicated by reduced respiratory exchange ratios (RER), lower ratings of perceived exertion (RPE), enhanced heart rate variability (HRV), and improved ventilatory efficiency. These findings highlight the importance of individualized rehabilitation approaches in optimizing the recovery process, enhancing cardiovascular health, and improving overall well-being in stroke survivors.

## Figures and Tables

**Table 1:** Demographic data of participants in Group A and Group B.

Variable	Group A	Group B	χ^2^ (Chi-Square)/ t Value	*p* Value
Age	59.60 ± 2.72	60.40 ± 3.20	−0.74	0.47
Height	1.47 ± 0.44	1.45 ± 0.43	1.22	0.23
Weight	65.20 ± 9.72	64.80 ± 10.21	0.11	0.91
BMI	30.84 ± 1.79	30.62 ± 4.12	0.19	0.85
Stroke type				
Ischemic	13 (86.7%)	11 (73.3%)	0.83	0.36
Hemorrhagic	2 (13.3%)	4 (26.7%)		
Side				
Right	11 (73.3%)	12 (80.0%)	0.19	0.67
Left	4 (26.7%)	3 (20.0%)		
Smoking				
Yes	10 (66.7%)	14 (93.3%)	3.33	0.17
No	5 (33.3%)	1 (5.7%)		
Hypertension				
Yes	10 (66.7%)	11 (73.3%)	0.16	0.69
No	5 (33.3%)	4 (26.7%)		
Diabetes				
Yes	14 (93.3%)	12 (80.0%)	1.15	0.28
No	1 (6.7%)	3 (20.0%)		
Heart Failure				
Yes	12 (80.0%)	11 (73.3%)	0.19	0.67
No	3 (20%)	4 (26.7%)		

**Table 2:** Independent *t*-test for comparative analysis of pre and post data between groups to assess variations in cardiovascular and autonomic nervous system parameters.

Variable		Group A Mean ± SD	Group B Mean ± SD	t-Value	*p*-Value	95% CI (Lower–Upper)
Mean NN	Pre	819.83 ± 62.12	834.72 ± 44.82	−0.75	0.46	−55.40–25.63
	Post	839.69 ± 52.72	910.82 ± 72.93	−3.06	0.01	−118.73–−23.54
Resting HR	Pre	75.27 ± 4.43	77.33 ± 6.31	−1.04	0.31	−6.15–2.01
	Post	72.47 ± 7.07	70.13 ± 6.45	0.95	0.35	−2.73–7.39
SDNN	Pre	137.94 ± 67.82	145.08 ± 60.40	−0.31	0.76	−55.18–40.88
	Post	153.16 ± 62.83	156.55 ± 62.17	−0.15	0.88	−50.14–43.37
RMSSD	Pre	33.79 ± 7.26	34.91 ± 7.59	−0.41	0.68	−6.67–4.44
	Post	40.54 ± 10.46	50.83 ± 9.80	−2.78	0.01	−17.87–−2.71
pNN50	Pre	42.52 ± 6.49	42.05 ± 5.05	0.22	0.83	−3.88–4.82
	Post	43.99 ± 6.19	44.68 ± 5.20	−0.33	0.75	−4.96–3.59
LF	Pre	398.73 ± 163.33	425.19 ± 139.34	−0.48	0.64	−140.01–87.09
	Post	361.03 ± 136.36	352.56 ± 159.72	0.16	0.88	−102.60–119.55
HF	Pre	288.85 ± 109.40	301.94 ± 127.05	−0.30	0.76	−101.77–75.59
	Post	346.13 ± 115.41	426.07 ± 153.52	−1.61	0.12	−181.52–21.64
VLF	Pre	170.98 ± 17.60	170.67 ± 9.75	0.06	0.95	−10.34–10.95
	Post	155.27 ± 18.56	159.85 ± 9.12	−0.86	0.40	−15.52–6.36
TP	Pre	903.13 ± 39.93	911.90 ± 44.54	−0.57	0.57	−40.41–22.87
	Post	915.19 ± 59.93	921.43 ± 70.68	−0.26	0.80	−55.25–42.77
LF/HF	Pre	1.46 ± 0.53	1.73 ± 1.09	−0.87	0.39	−0.91–0.37
	Post	1.10 ± 0.40	0.90 ± 0.43	1.27	0.21	−0.12–0.51
nLF	Pre	44.20 ± 18.06	47.01 ± 17.70	−0.43	0.67	−16.19–10.56
	Post	39.89 ± 16.12	38.57 ± 17.50	0.21	0.83	−11.27–13.90
nHF	Pre	32.08 ± 12.37	33.12 ± 13.84	−0.22	0.83	−10.86–8.78
	Post	38.02 ± 12.78	46.91 ± 18.70	−1.52	0.14	−20.87–3.09

Note: Mean NN (Mean of NN intervals), Resting HR (Resting Heart Rate), SDNN (Standard Deviation of NN intervals), RMSSD (Root Mean Square of Successive Differences), pNN50 (Percentage of NN intervals differing by more than 50 ms), LF (Low Frequency), HF (High Frequency), VLF (Very Low Frequency), TP (Total Power), LF/HF (Low Frequency to High Frequency Ratio), nLF (Normalized Low Frequency), nHF (Normalized High Frequency), MD (Mean Difference), SD (Standard deviation of mean difference), t (Statistical value of paired *t* test), *p* (significance value), CI (Confidence Interval).

**Table 3:** Independent *t*-test for comparative analysis of pre and post data between groups to assess variations in cardiovascular and respiratory parameters, exercise efficiency, perceived exertion, and exercise duration.

Variable		Group A Mean ± SD	Group B Mean ± SD	t-Value	*p*-Value	95% CI (Lower–Upper)
SBP (mmHg)	Pre	127.13 ± 14.01	125.80 ± 14.08	0.26	0.797	−9.173–11.839
	Post	122.73 ± 12.24	117.67 ± 15.12	1.01	0.322	−5.222–15.356
DBP (mmHg)	Pre	80.07 ± 6.11	79.80 ± 6.84	0.11	0.911	−4.583–5.117
	Post	77.87 ± 6.06	78.00 ± 7.28	−0.06	0.957	−5.142–4.876
HR Rest (bpm)	Pre	75.13 ± 15.31	77.93 ± 10.28	−0.59	0.561	−12.553–6.953
	Post	80.60 ± 9.92	70.47 ± 9.20	2.90	0.007	2.977–17.290
HR Peak bpm	Pre	101.40 ± 5.15	109.60 ± 6.25	−3.92	0.001	−12.486–−3.914
	Post	109.07 ± 8.05	115.27 ± 7.38	−2.20	0.036	−11.976–−0.424
VE Peak (L/min)	Pre	42.25 ± 12.11	41.38 ± 13.73	0.19	0.854	−8.806–10.561
	Post	50.81 ± 10.11	59.30 ± 10.99	−2.20	0.036	−16.389–−0.586
RR Peak (bpm)	Pre	20.60 ± 6.93	21.43 ± 4.77	−0.38	0.706	−5.274–3.621
	Post	18.31 ± 3.85	15.74 ± 4.00	1.79	0.084	−0.365–5.512
PET CO_2_ Rest (mmHg)	Pre	34.29 ± 5.42	32.83 ± 4.29	0.82	0.420	−2.196–5.116
	Post	32.85 ± 5.22	30.147 ± 2.95	1.75	0.091	−0.462- 5.876
PET CO_2_ Peak (mmHg)	Pre	43.32 ± 3.32	41.49 ± 3.35	1.50	0.144	−0.666–4.319
	Post	40.96 ± 3.56	33.77 ± 2.68	6.25	<0.001	4.832–9.541
VE/CO_2_ Slope	Pre	38.51 ± 3.83	39.12 ± 3.75	−0.44	0.661	−3.448–2.221
	Post	36.48 ± 3.59	32.24 ± 4.42	2.88	0.007	1.228–7.252
VE/O_2_ Slope	Pre	24.38 ± 4.85	23.58 ± 2.58	0.56	0.579	−2.108–3.708
	Post	21.85 ± 4.76	17.94 ± 4.47	2.32	0.028	0.453–7.355
RER	Pre	1.14 ± 0.14	1.09 ± 0.10	1.16	0.257	−0.039–0.142
	Post	1.10 ± 0.15	0.97 ± 0.16	2.36	0.026	0.017–0.249
Workload (watts)	Pre	85.15 ± 16.50	82.29 ± 7.87	0.61	0.550	−6.800–12.533
	Post	97.54 ± 15.53	93.49 ± 20.07	−0.62	0.541	−9365–−17.477
RPE	Pre	13.07 ± 2.34	14.23 ± 2.55	−1.29	0.208	−2.985–0.678
	Post	12.02 ± 2.53	11.36 ± 2.34	0.75	0.460	−1.155–2.488

Note: SBP (Systolic Blood Pressure), DBP (Diastolic Blood Pressure), HR Rest (Heart Rate at Rest), HR Peak (Peak Heart Rate), VE Peak (Peak Minute Ventilation), RR Peak (Peak Respiratory Rate), PET CO_2_ Rest (End-Tidal CO_2_ at Rest), PET CO_2_ Peak (Peak End-Tidal CO_2_), VE/CO_2_ Slope (Minute Ventilation/Carbon Dioxide Production Slope), VE/O_2_ Slope (Minute Ventilation/Oxygen Uptake Slope), RER (Respiratory Exchange Ratio), Workload (Workload in watts), RPE (Rate of Perceived Exertion), t (Statistical value of paired *t* test), *p* (significance value).

**Table 4:** Paired *t*-test comparing pre- and post-intervention changes in cardiovascular fitness and heart rate variability (HRV) parameters within Group A and Group B.

Variables	Group	Pre	Post	MD	SD	95% CI (Lower–Upper)	t	*p*
Mean NN	Group A	819.83 ± 62.12	839.69 ± 52.72	−19.86	49.37	−47.20–7.49	−1.56	0.14
	Group B	834.72 ± 44.82	910.82 ± 72.93	−76.10	69.23	−114.44–−37.76	−4.26	<0.001
Resting HR	Group A	75.27 ± 4.43	72.47 ± 7.07	2.80	4.97	0.05–5.56	2.18	0.05
	Group B	77.33 ± 6.31	70.13 ± 6.45	7.20	7.87	2.84–11.56	3.55	<0.001
SDNN	Group A	137.94 ± 67.82	153.16 ± 62.83	−15.23	29.13	−31.36–0.90	−2.03	0.06
	Group B	145.08 ± 60.40	156.55 ± 62.17	−11.47	15.64	−20.13–−2.81	−2.84	0.01
RMSSD	Group A	33.79 ± 7.26	40.54 ± 10.46	−6.75	12.42	−13.62–0.13	−2.11	0.05
	Group B	34.91 ± 7.59	50.83 ± 9.80	−15.92	9.03	−20.92–−10.92	−6.83	<0.001
pNN50	Group A	42.52 ± 6.49	43.99 ± 6.19	−1.48	1.14	−2.11–−0.84	−5.01	0.00
	Group B	42.05 ± 5.05	44.68 ± 5.20	−2.63	1.11	−3.24–−2.02	−9.19	<0.001
LF	Group A	398.73 ± 163.33	361.03 ± 136.36	37.70	247.53	−99.38–174.77	0.59	0.57
	Group B	425.19 ± 139.34	352.56 ± 159.72	72.63	214.69	−46.26–191.52	1.31	0.21
HF	Group A	288.85 ± 109.40	346.13 ± 115.41	−57.27	150.20	−140.45–25.91	−1.48	0.16
	Group B	301.94 ± 127.05	426.07 ± 153.52	−124.12	159.74	−212.58–−35.66	−3.01	0.01
VLF	Group A	170.98 ± 17.60	155.27 ± 18.56	15.71	27.47	0.50–30.92	2.21	0.04
	Group B	170.67 ± 9.75	159.85 ± 9.12	10.83	14.74	2.66–18.99	2.84	0.01
TP	Group A	903.13 ± 39.93	915.19 ± 59.93	−12.06	84.83	−59.04–34.92	−0.55	0.59
	Group B	911.90 ± 44.54	921.43 ± 70.68	−9.53	45.78	−34.88–15.82	−0.81	0.43
LF/HF	Group A	1.46 ± 0.53	1.10 ± 0.40	0.36	0.66	−0.00–0.73	2.14	0.05
	Group B	1.73 ± 1.09	0.90 ± 0.44	0.83	1.19	0.17–1.49	2.70	0.02
nLF	Group A	44.20 ± 18.06	39.89 ± 16.12	4.31	28.97	−11.73–20.35	0.58	0.57
	Group B	47.01 ± 17.71	38.57 ± 17.50	8.44	24.16	−4.94–21.82	1.35	0.20
nHF	Group A	32.08 ± 12.37	38.02 ± 12.78	−5.94	17.91	−15.86–3.97	−1.29	0.22
	Group B	33.12 ± 13.84	46.91 ± 18.70	−13.79	19.38	−24.53–−3.06	−2.76	0.01

Note: Mean NN (Mean of NN intervals), Resting HR (Resting Heart Rate), SDNN (Standard Deviation of NN intervals), RMSSD (Root Mean Square of Successive Differences), pNN50 (Percentage of NN intervals differing by more than 50 ms), LF (Low Frequency), HF (High Frequency), VLF (Very Low Frequency), TP (Total Power), LF/HF (Low Frequency to High Frequency Ratio), nLF (Normalized Low Frequency), nHF (Normalized High Frequency), MD (Mean Difference), SD (Standard deviation of mean difference), t (Statistical value of paired *t* test), *p* (significance value), CI (Confidence Interval).

**Table 5:** Paired *t*-test results for pre- and post-intervention changes in cardiovascular and respiratory parameters, exercise efficiency, perceived exertion, and exercise duration.

Variables	Group	Pre	Post	MD	SD	95% CI (Lower–Upper)	t	*p*
SBP (mmHg)	Group A	127.13 ± 14.01	122.73 ± 12.24	4.4	8.92	−0.54–9.34	1.91	0.08
	Group B	125.80 ± 14.08	117.67 ± 15.12	8.13	5.45	5.12–11.15	5.78	<0.001
DBP (mmHg)	Group A	80.07 ± 6.11	77.87 ± 6.06	2.2	4.2	−0.12–4.52	2.03	0.06
	Group B	79.80 ± 6.84	78.00 ± 7.28	1.8	3.49	−0.13–3.73	1.99	0.07
HR Rest (bpm)	Group A	75.13 ± 15.31	80.60 ± 9.92	−5.47	16.89	−14.82–3.89	−1.25	0.23
	Group B	77.93 ± 10.28	70.47 ± 9.20	7.47	10.51	1.65–13.29	2.75	0.02
HR Peak (bpm)	Group A	101.40 ± 5.15	109.07 ± 8.05	−7.67	8.93	−12.61–−2.71	−3.32	0.01
	Group B	109.60 ± 6.25	115.27 ± 7.38	−5.67	8.64	−10.45–−0.88	−2.54	0.02
VE Peak L/min	Group A	42.25 ± 12.11	50.81 ± 10.11	−8.56	18.49	−18.8–1.68	−1.79	0.10
	Group B	41.38 ± 13.73	59.30 ± 10.99	−17.93	20.71	−29.39–−6.46	−3.35	0.01
RR Peak (bpm)	Group A	20.60 ± 6.93	18.31 ± 3.86	2.29	8.03	−2.16–6.74	1.1	0.29
	Group B	21.43 ± 4.77	15.74 ± 4.00	5.69	4.99	2.92–8.45	4.41	<0.001
PET CO_2_ Rest (mmHg)	Group A	34.29 ± 5.42	32.85 ± 5.22	1.44	5.31	−1.5–4.38	1.05	0.31
	Group B	32.83 ± 4.29	30.15 ± 2.95	2.69	5.39	−0.29–5.67	1.93	0.07
PET CO_2_ Peak (mmHg)	Group A	43.32 ± 3.32	40.96 ± 3.56	2.36	4.17	0.05–4.67	2.19	0.05
	Group B	41.49 ± 3.35	33.77 ± 2.68	7.72	4.04	5.48–9.96	7.4	<0.001
VE/CO_2_ Slope	Group A	38.51 ± 3.83	36.48 ± 3.59	2.03	5.19	−0.85–4.9	1.51	0.15
	Group B	39.12 ± 3.75	32.24 ± 4.42	6.88	1.56	6.01–7.74	17.13	<0.001
VE/O_2_ Slope	Group A	24.38 ± 4.85	21.85 ± 4.76	2.53	4.81	−0.13–5.2	2.03	0.06
	Group B	23.58 ± 2.58	17.94 ± 4.47	5.64	5.81	2.42–8.85	3.76	<0.001
RER	Group A	1.14 ± 0.14	1.10 ± 0.15	0.04	0.05	0.01–0.07	3.06	0.01
	Group B	1.09 ± 0.10	0.97 ± 0.16	0.12	0.18	0.02–0.22	2.63	0.02
Workload (watts)	Group A	85.15 ± 16.50	97.54 ± 15.53	−12.39	23.12	−25.19–0.42	−2.07	0.06
	Group B	82.29 ± 7.87	93.48 ± 20.07	−11.2	20.12	−22.34–−0.05	−2.16	0.05
RPE	Group A	13.07 ± 2.34	12.02 ± 2.53	1.05	3.94	−1.13–3.23	1.03	0.32
	Group B	14.23 ± 2.55	11.36 ± 2.34	2.87	3.04	1.18–4.56	3.65	<0.001

Note: SBP (Systolic Blood Pressure), DBP (Diastolic Blood Pressure), HR (Heart Rate), VE (Minute Ventilation), RR (Respiratory Rate), CO_2_ (Carbon dioxide), O_2_ (oxygen), PET (End-Tidal Pressure), VE/CO_2_ (Ventilatory Equivalent for CO_2_), RER (Respiratory Exchange Ratio), RPE (Rate of Perceived Exertion).

## Data Availability

The data presented in this study are available on request from the corresponding author. The data are not publicly available due to privacy restrictions.
